# TiO_2_–LiF composite coating for improving NCM622 cathode cycling stability: one-step construction†

**DOI:** 10.1039/d3ra05659g

**Published:** 2023-11-20

**Authors:** Kai Huang, Jinxia Zhou, Huili Yang, Tianzheng Xie, Tu Lan, Suichang Ong, Heng Jiang, Yibo Zeng, Hang Guo, Ying Zhang

**Affiliations:** a Pen-Tung Sah Institute of Micro-Nano Science and Technology, Xiamen University Xiamen Fujian 361005 People's Republic of China hangguo@xmu.edu.cn; b College of Chemistry and Chemical Engineering, Xiamen University Xiamen Fujian 361005 People's Republic of China yzhang@xmu.edu.my; c Xiamen University Malaysia 43900 Sepang Selangor Daryl Ehsan Malaysia

## Abstract

The Ni-rich NCM622 is a promising cathode material for future high energy lithium ion batteries, but unstable electrochemical performance of NCM622 hinder its large scale commercial application. The cycling peformance of nickel-rich LiNi_0.6_Co_0.2_Mn_0.2_O_2_ (NCM622) cathode materials can be improved by surface coating. Here, a one-step approach based on TiF_4_ is used to successfully manufacture modified NCM622 cathode materials with a TiO_2_–LiF coating. The TiO_2_–LiF coated NCM622 preserves 79.7% capacity retention which is higher than the pure NCM622 (68.9%) at 1C after 200 cycles within 2.7–4.3 V. This material serves as the cathode for lithium-ion batteries (LIBs). The uniform TiO_2_–LiF coating layer can alleviate structural degradation brought on by unfavorable side reactions with the electrolyte has been validated. TiO_2_–LiF coated on NCM622 cathode materials can be modified easily by one-step approach.

## Abstract 2

The cycling performance of nickel-rich LiNi_0.6_Co_0.2_Mn_0.2_O_2_ (NCM622) cathode of lithium-ion batteries (LIBs) can be improved by TiO_2_–LiF surface coating.

## Graphical abstract

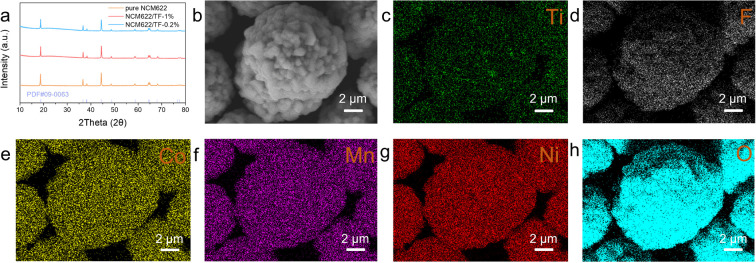

## 1. Introduction

Rechargeable lithium-ion batteries (LIBs) have been widely used in electric vehicles due to the demand for reliable energy storage solutions from society.^1–4^ The cycling performances and safety of LIBs are significantly influenced by cathode materials.^5,6^ To fulfill the increased energy demands, it is imperative to research and develop high capacity, superior stability and low-cost cathode materials. Nickel-rich LiNi_*x*_Co_*y*_Mn_*z*_O_2_ (NCM, *x* ≥ 0.6) cathode materials have drawn a lot of interest due to its high specific capacity and low cost.^7^ Among them, the LiNi_0.6_Co_0.2_Mn_0.2_O_2_ (NCM622) cathode materials offer higher reversible capacity and superior cycling stability owing to its low Ni concentration. Therefore, NCM622 cathode material is a promising candidate for high-energyLIBs.^8,9^

Raising the cut-off voltage can increase the discharge capacity of NCM622 cathode materials. However, the irreversible phase transition of NCM622 at high charging voltage results in the destruction of the surface structure of NCM622.^10,11^ Additionally, unintentionally generated side reactions between NCM622 and the electrolyte, such as the development of cathode electrolyte interphase (CEI) and the dissolution of transition metal ions, may reduce the active components and reversible capacity. Surface lithium residues on NCM622 like LiOH and Li_2_CO_3_ have an impact on the increased impedance and rapid capacity fading_._^12^ Higher Ni content of NCM622 causes oxygen evolution and surface degradation at higher voltage, which also hastens the decline in cycling performance.^13^

Electrolyte engineering has improved the structural stability of the Ni-rich cathode surface by creating LiF-rich and F-rich interphases on the cathode surfaces, a robust CEI with fluoride (F) and boron (B) by using LiDFOB additive, a Li_3_PO_4_ and LiF CEI using LiPF_6_ as a cathode additive, and using PFPN-based electrolyte to form a LiF rich CEI.^14–17^ From the standpoint of scalability, repeatability, and flexibility of the material synthesis, rational composition design using elemental doping is considered to be the most practical way.^18,19^ The usage of other common dopants, including Mg, Zr, and Ti, is often rigorously regulated to below 1% among the various dopants.^20–22^ Surface coating is thought to be an efficient way to reduce side reactions at the CEI. The coating layer acting as a “protective bar” to prevent the side reaction of the cathode materials and the electrolyte, metal oxides has proven to be highly stable under demanding circumstances including working at wide potential window and cycling at higher temperature.^23–33^ To achieve a compromise between physical shielding and adequate charge transfer, metal oxides need to control coating thickness. Since the growth mechanisms of various materials in liquid are quite complex, not to mention the chemical reaction between the NCM622 and solvents, it is difficult to produce an evenly distributed and continuous layer with precise thickness using wet-chemistry processes like sol–gel and precipitation methods.^34,35^ With the exception of cost restrictions, gas phase technologies like chemical vapor deposition (CVD)^36,37^ and atomic layer deposition (ALD)^38–40^ are preferable options. Contrarily, despite its inadequacies in homogeneity, ball-milling for producing coating layer is available for industrial manufacturing and is not detrimental to the host material.^41,42^

In this study, we offer an easy wet-chemical method based on TiF_4_ for a composite coating of TiO_2_ and LiF that modifies the NCM622 surface. After annealing at a high temperature, a composite coating with a high degree of chemical stability was created on the NCM surface. TiO_2_ coating can stabilize the structure on the outer layer of NCM622 to a certain degree. In order to shield the NCM structure, inhibit side reactions of the electrolyte and the electrode, and offer active sites for quick transfer of Li^+^ and electrons, it is possible to use the TiO_2_ and LiF for CEI formation. This overcomes the disadvantages of inconsistent distribution and excessive thickness. The electrochemical performances have been significantly enhanced thanks to the successful *in situ* construction of a thin homogeneous TiO_2_–LiF layer on the surface of the host material through heat decomposition. At 2.7–4.3 V at 1C, the electrochemical properties of NCM622 were evaluated, then the mechanism of improvement was investigated.

## 2. Experimental section

### 2.1 Preparation of TF-NCM622

#### 2.1.1 Synthesis of materials

Commercial NCM622 was bought from Canard, while supplies for TiF_4_ (99%) powder were bought from Aladdin. To create a consistent solution with a certain concentration, TiF_4_ is first dissolved in anhydrous ethanol. After that, the appropriate quantity of NCM622 powder had been added to the solution in accordance with the predetermined weight ratio (TiF_4_ : NCM622 = 0.2% or 1%). The mixture had a 30 minute ultrasonic treatment before being moved to an oil-bathing pot, immediately agitated at 60 °C until it got viscous, subsequently removed, evaporated in a vacuum oven for 10 hours, followed by calcined in the air at 400 °C for an hour to produce the finished product. The labels on the goods read NCM622/TF-0.2% and NCM622/TF-1%.

### 2.2 Electrode preparation and cell assembly

PVDF 5130, super P and NCM622 (1 : 1 : 8, wt%) are dissolved in *N*-methyl-2-pyrrolidone (NMP) to prepare the NCM622/TF electrode slurry. The NMP was removed by casting the resulting slurry on a foil of aluminum and drying it at 110 °C overnight under vacuum. The NCM622 weight loading was 3.4 mg cm^−2^ after disc electrodes with a diameter of 10 mm were pierced. Lithium foil serving as anodes, a Celgard 2500 membrane serving as the separator, and ethylene carbonate (EC), dimethyl carbonate (DMC), and diethyl carbonate (DEC) (v/v/v = 1 : 1 : 1) serving as the electrolyte. The electrolyte volume is 60 μL added to the coin cell.

### 2.3 Materials characterization

The sample's crystal phase was identified using a technique known as X-ray diffraction (XRD, Rigaku Ultima IV). The scanning electron microscopy technique (SEM, ZEISS MERLIN Compact) was used to analyze the sample's morphology, and a dispersive spectrometer with energy dispersive (EDS, FEl-inspect-F50) was used to identify the distribution of the elements. Using a TEM (Talos F200s), the shape and structure of the lattice of the coated layer were investigated. The composition of its surface was investigated using XPS (Thermo Kalpha, XPS), an X-ray photoelectron spectroscopy technique.

### 2.4 Electrochemical measurements

The electrochemical impedance spectroscopy (EIS) and cyclic voltammetry (CV) measurements performed on an CHI660E electrochemical workstation. Prior to the cycle, the EIS was assessed at 3.0 V of voltage, 10^−2^ to 10^5^ Hz of scanning frequency, and 5 mV of amplitude.

## 3. Results and discussion

**Fig. 1:**
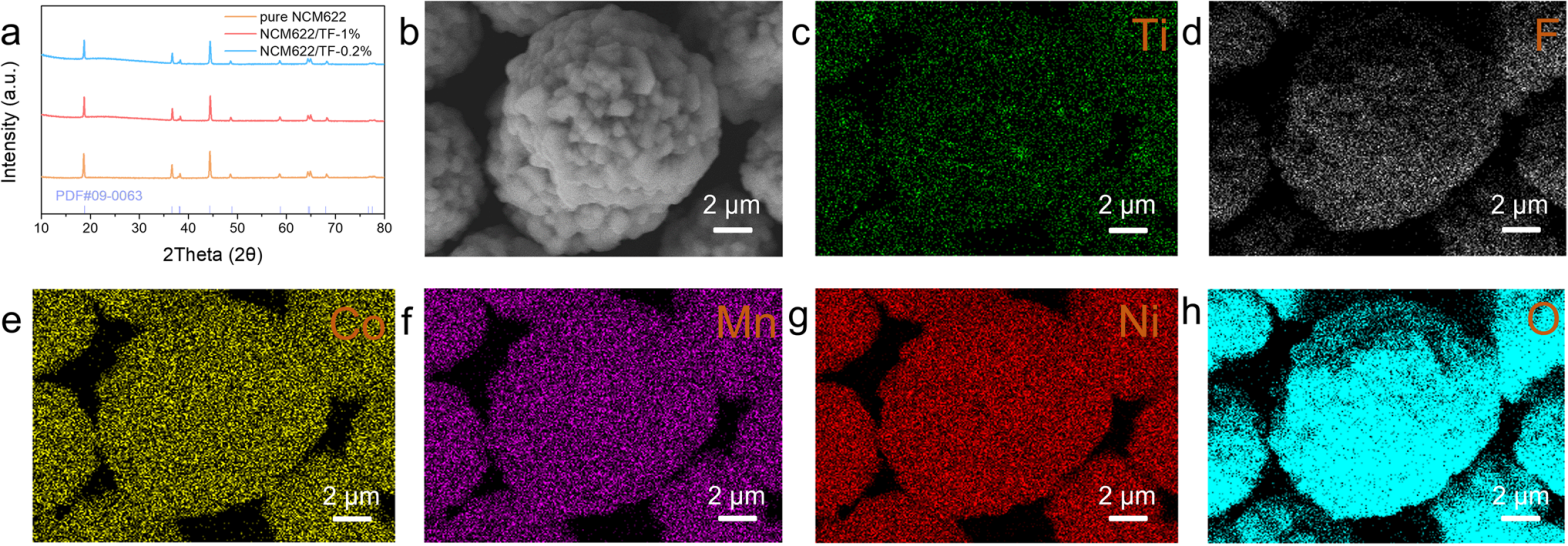
(a) XRD patterns of pure NCM622, NCM622/TF-0.2% and NCM622/TF-1% sample. (b–h) SEM images of and the EDS mapping of NCM622/TF-0.2%.

Fig. 1a displays the XRD patterns for samples of coated and pure NCM622. Since there are no extra second-phase diffraction peaks in any of the samples, the coating concentration is quite low. The peak intensities of the NCM622/TF-0.2% and NCM622/TF-1% samples were comparable to those of pure NCM622, but the (003) peak values of the NCM622/TF-0.2% and NCM622/TF-1% samples are poor, possibly as a result of the thick coating obstructing the equipment detection signal. The NCM622 surface was covered with a thin layer of TiF_4_ using a wet chemical process, and during high-temperature solid-state sintering, TiF_4_ gradually changed into TiO_2_. The impact of the composite barrier has been predicted theoretically as Li atoms presented on the NCM622 surfaces reacting with gaseous fluoride compounds broken down in TiF4 to generate LiF. SEM was used to examine the morphology of samples following alteration (Fig. 1b). The elements Ti, F, O, Ni, Co, and Mn are evenly dispersed throughout NCM622 according to the EDS-mapping of NCM622/TF-0.2%.

**Fig. 2:**
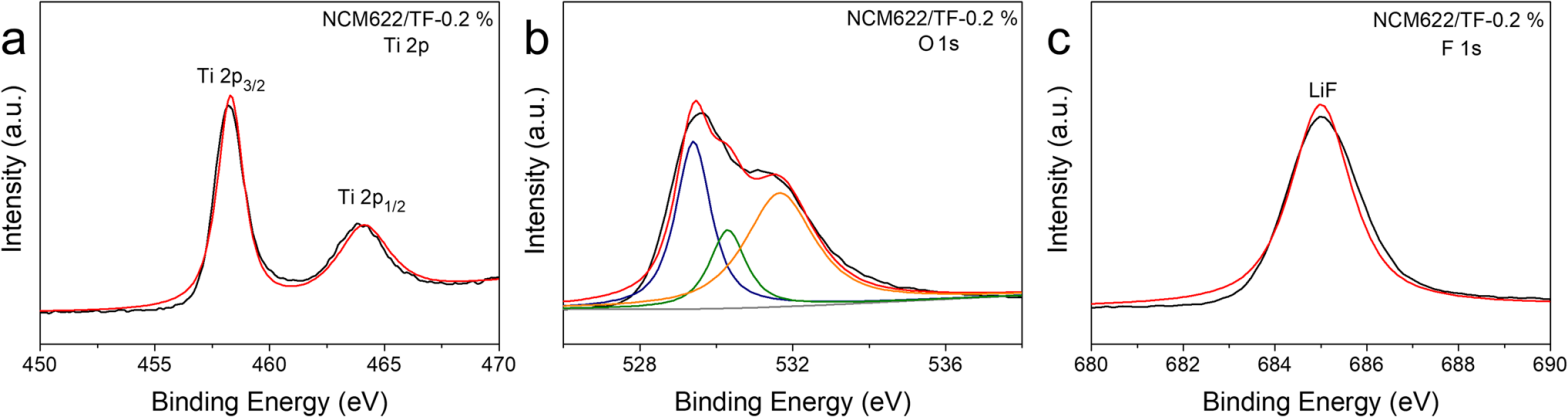
(a–c) XPS spectrum of NCM622/TF-0.2% sample, and XPS scans of Ti 2p, O 1s and F 1s.

The samples that were collected before and after wrapping were subjected to XPS testing in order to further verify the presence and chemical makeup of the coating.^43,44^ The entire XPS spectrum (Fig. 2) shows peaks of Ti 2p, O 1s, and F 1s. The findings demonstrate that F and Ti do not totally evaporate and vanish after being calcined at 400 °C; rather, they continue to cling to NCM622's surface. In the Ti 2p spectra (Fig. 2a), the outer layer of the pure NCM622 sample did not exhibit any Ti signal, but the coated NCM622/TF-0.2% samples surface did exhibit the typical Ti 2p orbital peak spectrum. Both orbital peaks correspond to Ti^4+^ in TiO_2_, and signal peaks of Ti 2p_3/2_ and Ti 2p_1/2_ were seen at 458.3 and 464.3 eV, respectively. The essential signal peak of NCM622 lattice oxygen is located at 529 eV in the O 1s spectrum (Fig. 2b). The oxygen vacancy on the surface of NCM622 is the cause of the oxygen adsorption peak at 531.3 eV. It can be noted that the NCM622/TF-0.2% sample's oxygen vacancy peaking/lattice oxygen peak intensity was weaker than the pure NCM622, indicating the number of oxygen vacancies in the topmost layer of the NCM622 after its coating was reduced, which is advantageous to the device. The Ti–O bond in the TiO_2_ compound is represented by the yellow peak at 529.7 eV, which shows that the TiF_4_ was converted into the TiO_2_ at a high temperature. The signal in the F 1s spectra that corresponds to the peak of elemental F was also exclusively seen on the surface of the NCM622/TF-0.2% sample (Fig. 2c). The peak at 685.0 eV is attributable to LiF produced on the NCM622 surface, further demonstrating that Li exposure on the surface layer during the high-temperature heat treatment reacted with the gaseous fluorine compound dissolved by TiF_4_. The aforementioned findings demonstrate that the TiO_2_–LiF coating applied to the NCM622 surface within this study was successfully created.

**Fig. 3:**
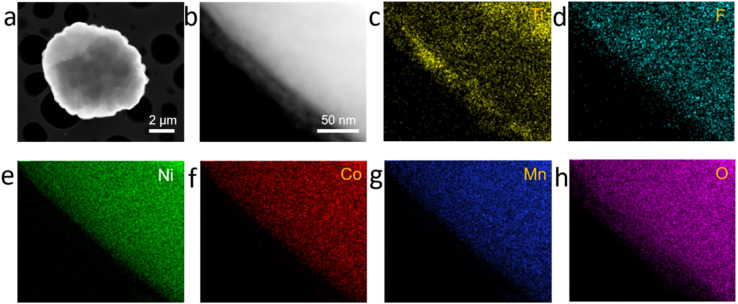
(a and b) TEM images of the NCM622/TF-0.2% sample. (c–h) EDX mapping of NCM622/TF-0.2% sample.

The coated NCM622 was characterized by HR-TEM techniques and EDX-mapping in order to further observe the structure of the microstructure and external element distribution. As can be seen in Fig. 3a and b, the coating of the NCM622 particles has been applied, and it is largely finished. The coating is distinct from the NCM622 structure. On NCM622/TF-0.2% and NCM622/TF-1%, the coating layer thickness is approximately 10 nm (Fig. 3a) and 43 nm (ESI, Fig. S1†). While 45 nm will disrupt the electron and ion transport of NCM622 particles, which will impair the cycle stability and surface impedance, 10 nm of TiO_2_–LiF is a more acceptable coating layer thickness. The edge coating layer's crystal orientation was distinct from the lattice stripes of the NCM622 as can be shown in the high-resolution TEM pictures (Fig. 3b). In the electron diffraction (EDS) spectrum (Fig. 3d–h), in addition to the signals from the elements Ni, Co, Mn, and O present throughout the NCM622 material, signals from the elements F and Ti were also discovered and evenly dispersed onto the NCM622 surface, demonstrating the effectiveness of the experimental coating.

**Fig. 4:**
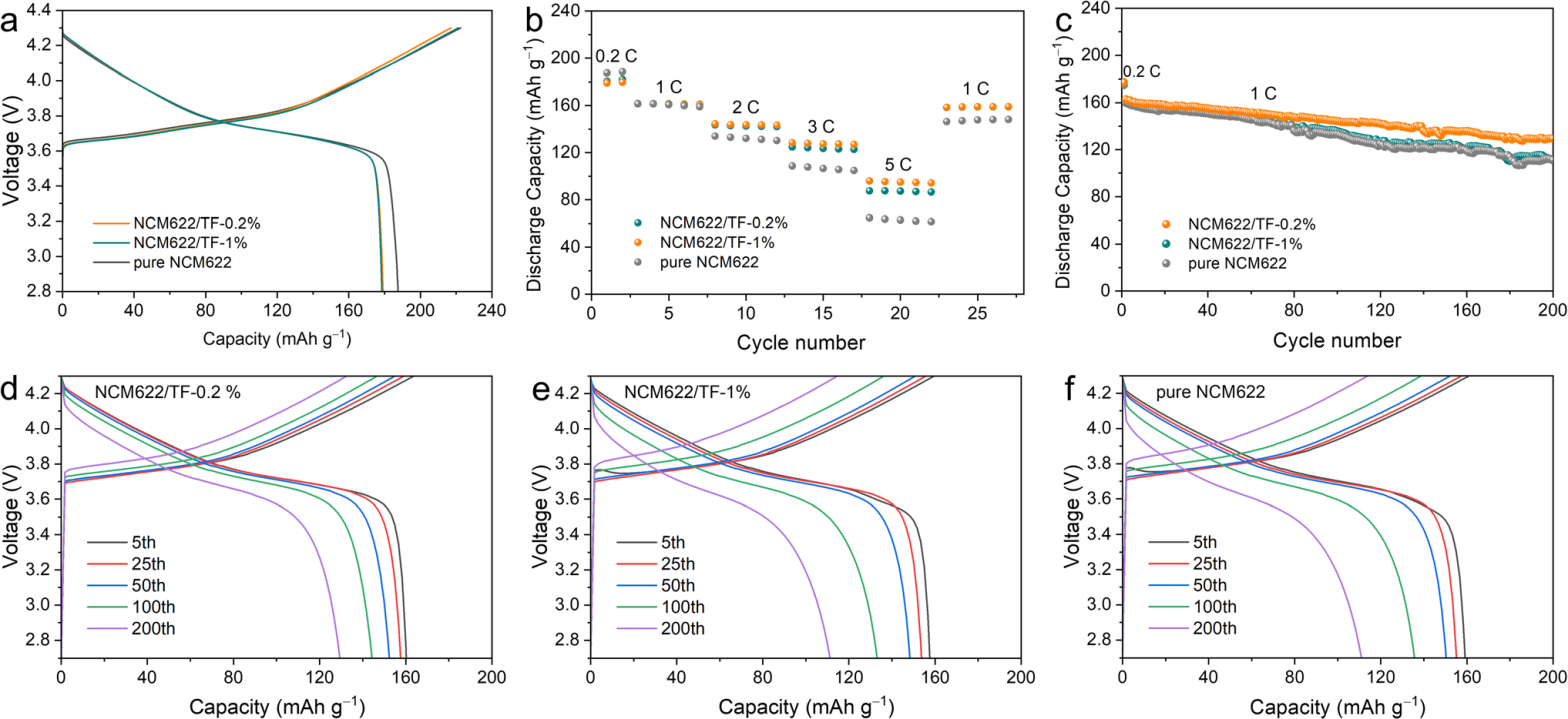
(a) Initial cycle curve, (b) rate performance, (c) long-term cycle and (d–f) cycle curve of the pure NCM622, NCM622/TF-0.2% and NCM622/TF-1% cathodes.

A charge–discharge measurement of the NCM622/Li half-cell had been carried out at 2.7–4.3 V and 1C (1C = 180 mA h g^−1^) to confirm the impact of the TiO_2_–LiF layer on the electrochemical properties of NCM622. The results are given in Fig. 4a. At 0.2C, the initial coulombic efficiency (CE) of pure NCM622, NCM622/TF-0.2%, and NCM622/TF-1% is 84.5%, 82.5%, and 80.71%, respectively. Since the materials' cycle curves are comparable, it can be assumed that the specific capacity is hardly affected by the TiO_2_–LiF coating. The discharge capacity value for both materials at different rates is shown in Fig. 4b. The discrepancy between the discharge specific capacities grows as the discharge current density rises. The capacities of the pure NCM622, NCM622/TF-0.2%, and NCM622/TF-1% were 64.7, 87.5, and 155.7 mA h g^−1^, respectively, as the current density increased to 5C, with the gap continuously widening. Due to the optimized lithium-ion migration circumstances, which boosted the lithium-ion transfer rate and encouraged the liberation of its capacity, the original capacity of coated NCM622 was not significantly lowered. Additionally, the surface side reaction that produced CEI, which immediately consumed the active components and decreased the initial discharging capacity of unmodified NCM622, has an impact on the discharge capacity. A crucial element of CEI, the LiF that forms on the outer layer of NCM622 after coating can prevent hazardous negative interactions between NCM622 and the electrolyte, lower the increase in charge transfer impedance, lower the loss of the chemically active lithium, and increase capacity of NCM622.

**Fig. 5:**
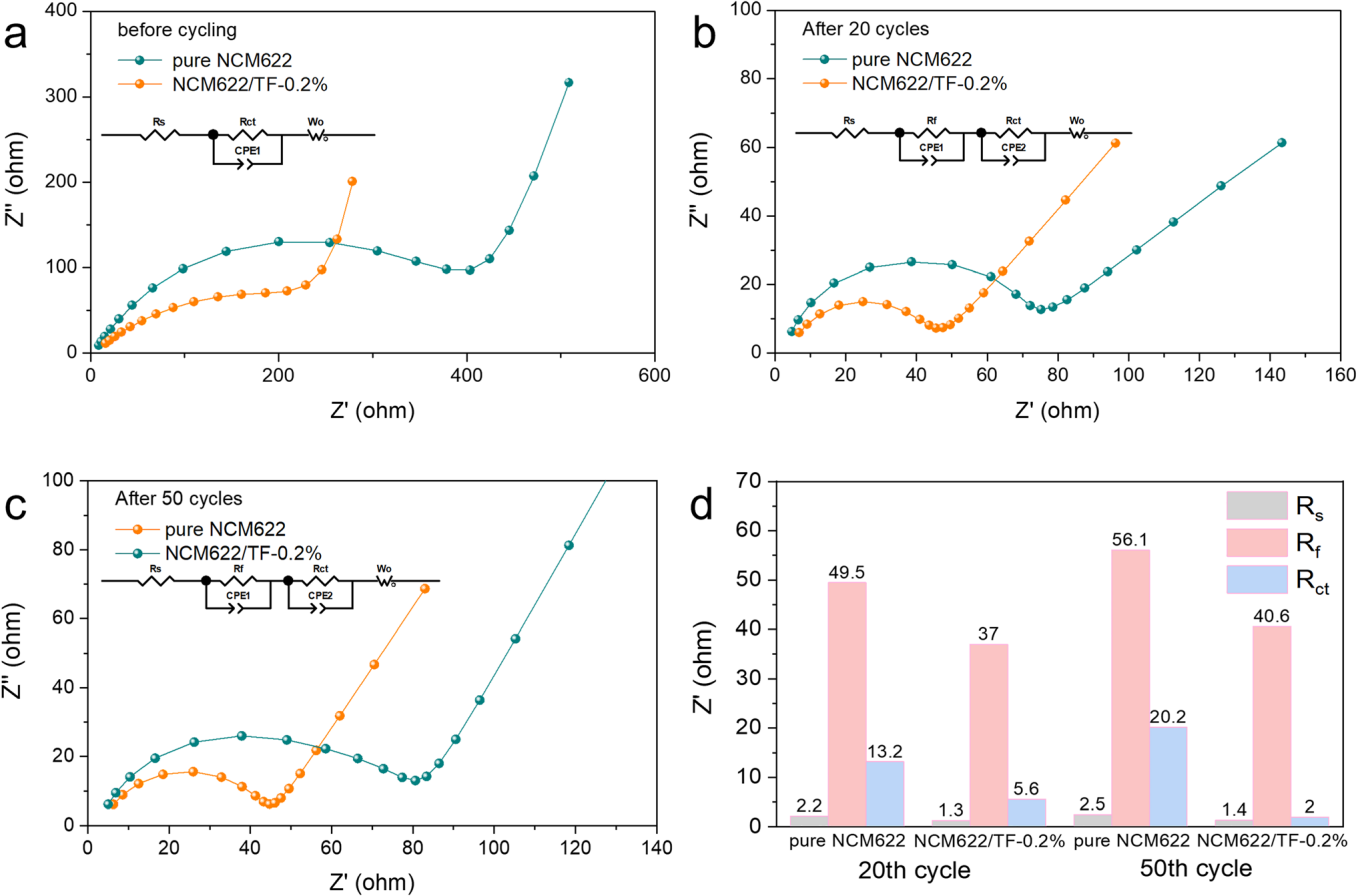
(a–c) Fitted EIS Nyquist plots of the pure NCM622 and NCM622/TF-0.2% electrodes before cycling, after 20 cycles and after 50 cycles. (d) Impedance histogram fitted to EIS curve.

To study the kinetics of the coated NCM 622 cathode material, EIS was recorded on pure NCM622 and NCM622/TF-0.2% before cycling, after 20 and 50 cycles, respectively. The model in Fig. 5 was used to fit the data. The resistance of CEI film (*R*_f_) and electron transfer impedance (*R*_ct_) is shown in the Nyquist plots. *R*_ct_ denotes the interfacial reactions for Li^+^ migration on the NCM622 surface. The *R*_s_ impedance values of pure NCM622 and NCM622/TF-0.2% have little change in different cycles. After 20 and 50 cycles, *R*_f_ decreased from 49.5 Ω to 37 Ω and from 56.1 Ω to 40.6 Ω, respectively, indicating that the coating improved the formation of CEI film. The *R*_ct_ of pure NCM622 becme larger at different cycles, which suggests that product of the side reation greatly obstructs Li^+^ transfer. While for NCM622/TF-0.2%, the Rct decreased after different cycles, it exhibits that the TiO_2_-LiF coating layer prevents HF corrosion of the electrode while impeding redox interactions between NCM622 and the electrolyte. The pure NCM622 sample's retention rate was only 68.9% (111.0 mA h g^−1^), the NCM622/TF-0.2% sample's capacity retention rate was 79.7% (129.4 mA h g^−1^), indicating that the TiO_2_–LiF hybrid coating still had an effective protective function on the NCM622.

## 4. Conclusion

In this study, a TiO_2_–LiF composite coating was created by heating NCM622 in one step with TiF_4_. The coated NCM622 demonstrated outstanding cycling performance. The NCM622/TF-0.2% exhibited excellent electrochemical performance, and demonstrated an retained capacity rate of 79.7% after 200 cycles in a voltage range of 2.7–4.3 V at 1C, pointing to a potential for future commercial application. This work demonstrates that the TiO_2_–LiF coating effectively prevents corrosion of the NCM622 bulk components by damaging chemicals in an electrolyte, strengthens the surface interior structure of the NCM622, and significantly alleviate the structural deterioration of the NCM622.

## Conflicts of interest

There are no declared conflicts.

## Supplementary Material

RA-013-D3RA05659G-s001
