# Supplementary material for: TiO2–LiF composite coating for improving NCM622 cathode cycling stability: one-step construction
Source: RSC Adv. 2023 Nov 20;13(48):33905–10. doi: 10.1039/d3ra05659g (PMC10658221; doi:10.1039/d3ra05659g)
Supplement: RA-013-D3RA05659G-s001 [file RA-013-D3RA05659G-s001.pdf]

Electronic Supplementary Material (ESI) for RSC Advances.
This journal is © The Royal Society of Chemistry 2023

Supporting Information

TiO_2-LiF Composite Coating for Improving NCM622 Cathode Cycling Stability: One-Step Construction

Kai Huang,[a] Jinxia Zhou,[a] Huili Yang,[a] Tianzheng Xie,[a] Tu Lan,[a] Suichang Ong,[ab] Heng Jiang,[a] Yibo Zeng,[a] Hang Guo,[*a] and Ying Zhang,[*bc]

a Pen-Tung Sah Institute of Micro-Nano Science and Technology, Xiamen University, Xiamen, Fujian 361005, People's Republic of China

b College of Chemistry and Chemical Engineering, Xiamen University, Xiamen, Fujian 361005, People's Republic of China

c Xiamen University Malaysia, 43900, Sepang, Selangor Daryl Ehsan, Malaysia

E-mail: hangguo@xmu.edu.cn and yzhang@xmu.edu.my

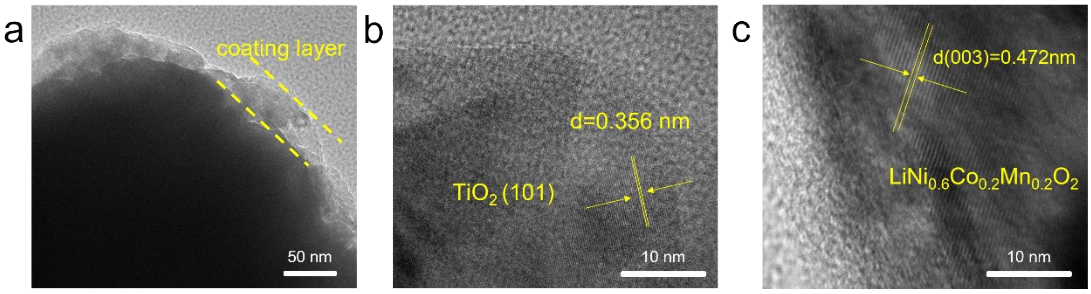

Figure S1. (a-c) HRTEM images of NCM622/TF-0.2%.
